# Governance of Iranian Primary Health Care System: Perceptions of Experts

**Published:** 2019-03

**Authors:** Jafar Sadegh TABRIZI, Faramarz POURASGHAR, Raana GHOLAMZADEH NIKJOO

**Affiliations:** 1. Health Services Management Research Center, Tabriz University of Medical Sciences, Tabriz, Iran; 2. Department of Health Services Management, School of Management and Medical Informatics, Tabriz University of Medical Sciences, Tabriz, Iran; 3. Road Traffic Injury Prevention Research Center, Tabriz University of Medical Sciences, Tabriz, Iran; 4. Department of Health Services Management, School of Management and Medical Informatics, Tabriz University of Medical Sciences, Tabriz, Iran; 5. Health Services Management Research Center, Iranian Center of Excellence in Health Management, Tabriz University of Medical Sciences, Tabriz, Iran; 6. Department of Health Services Management, School of Management and Medical Informatics, Tabriz University of Medical Sciences, Tabriz, Iran

**Keywords:** Clinical governance, Hospital specialists, Primary health care, Survey, Iran

## Abstract

**Background:**

Despite huge advances in improving most health indicators, Iranian primary health care (PHC) has faced several problems in improving the quality of care inside the health care system. Developed countries with similar problems have used various models of PHC governance for improving quality in their PHC system. This study aimed to obtain health professionals’ perspectives about the suitable pillars and components of Iran’s PHC governance model.

**Methods:**

A purposeful sampling method was used to select seven participants who had a minimum of five years of experience in PHC and background education in the field of medical sciences. Between Jan and Jun 2015, three focus group discussions (FGD) were conducted with seven PHC experts in Tabriz. Data were analyzed using the conventional content analysis method.

**Results:**

The eight main categories including quality improvement, management and leadership, community involvement and customer participation, effectiveness of PHC, human resource development, safety, health care evaluation and audit, and health information management plus 51 sub-categories were identified according to participants’ expects about the essential pillars and components for Iranian PHC governance model.

**Conclusion:**

Pillars that suggested for designing Iran’s PHC governance model are presented according to internal informed expert’s opinions and taking into account PHC system real status. By adding the degree of importance for each component and proper performance indicators to this collection, assessing the progress of the PHC system towards excellence will be possible and it will prevent any mental judgments about system performance.

## Introduction

The primary health care (PHC) approach, is a philosophy that contains principles and strategies for organizing health systems (1). Well-designed PHC is the foundation of any high-performance health care system to achieve better health care outcomes with the lowest cost (2). Demand for PHC services is expected to grow up due to changes like rising complex chronic diseases, increase in the elderly population with numerous comorbidities, manpower shortage, geographical dispersion, increasing health costs, and the development of new technologies in recent years (3). Hence, many countries have been focused on improving the quality of PHC (4). Quality improvement refers to tasks which aim to improve care and dissuade poor care on a continuous basis as part of daily services (5). North American countries, the United Kingdom (UK), Australia and New Zealand are the main examples of successful quality improvement in primary health care (6, 7).

Clinical governance is one of the important approaches to improve quality of health care systems. The term “clinical governance” describes a wide range of policies used to reform the National Health Service (NHS) in the UK (8). Aims of using clinical governance in PHC are to raise patient satisfaction, improve collaborative relationships and efficiency within and across clinical teams, increase job satisfaction for professionals, improve clinical outcomes and reduce adverse events (9, 10).

Around 1974, Iran began to study developing health systems. The first signs of the establishment of PHC appeared in 1979, but the full deployment of health care networks occurred in 1985. Following the establishment of the primary health care network in Iran, significant improvements occurred in some health indicators such as life expectancy, maternal mortality and child mortality (11).

In Iran, like other countries, improving the quality of health services is a high priority, and in order to achieve this goal, the implementation of clinical governance at the hospital level started in 2009. However its implementation was not successful; the implementation of clinical governance needs to have a bottom-up view and without improving the quality of services at the primary levels of the system (like the PHC level) it cannot be expected that an improvement in quality will result in the higher levels of service, such as hospitals (12,13). Therefore, the aim of this study was to obtain the perspective of health professionals about the elements and components of the PHC governance model for Iran.

## Materials and Methods

### Sampling and data collection

Between Jan and Jun 2015, three focus group discussions (FGD) were conducted with seven PHC experts in Tabriz. Participants were selected using purposive sampling. Selection of participants was on the basis of previous experience and current position at the universities and hospitals. Participants of this study were active in decision-making positions and as executive and academic staff inside health system and participated at different regional and national health levels. The inclusion criteria were as follows: minimum of five years of experience in PHC, background education in the field of medical sciences and the ability to express their opinions. The lack of tendency to participate in the study and revoking conscious satisfaction were considered as the exclusion criteria.

Those agreed to participate in focus group discussion sessions signed a consent form prior to their contribution. This study was approved by Tabriz University of Medical Sciences Ethics Committee (3045/77/5).

All FGDs were performed and recorded on a digital voice recorder after receiving permission from the participants. Each session lasted between 40–75 min (Mean=60′). Before starting FGDs, researchers explained the study objectives and reasons for their selection of FGD and clarified benefits of the study and how they could access the results of the study. FGDs were held for questions requiring open-ended and interpretative answers. The key question for the FGDs was: “What pillars and components for the Iranian model of PHC governance are essential?”

The semi-structured interview schedule is available in Table 1. FGD sessions were continued until findings were becoming repetitive. Finally, by the 3rd FGD, we reached data saturation. Notes were also taken during all FGDs and these recordings were fully transcribed and augmented by observational field notes.

**Table 1:** Semi structure interview schedule used in focus group discussions

***Main question***	***Additional question***	***Clarifying question***
What are the most important strengths and challenges of Iran’s primary health care system?	Have you noticed any changes in the situation of Iran’s primary health care in recent years	Can you give me some examples?
How is position of quality in Iran’s Primary Health Care system?	Whether in design or in subsequent amendments of Iran’s primary health care system are intended a place to improve the quality of it?	Can you expand a little on this?
What pillars and components for Iranian model of PHC governance are essential?	Why?	-
Do you have specific implementation practices for primary health care governance model?	-	Can you tell me anything else?

### Data analysis and interpretation

All crude data were firstly gathered in Persian language. Data collection and analysis were done simultaneously for 6 months in 2015. The MAXQDA10 software was used for transcription and classification of codes. Conventional content analysis was used to identify categories and themes. All of the researchers participated in the data coding. The process of sharing the data between researchers helped to improve the trustworthiness of the analysis and interpretation of the data. In addition, draft reports were sent back to each participant for respondent validation.

To ensure credibility, dependability, and conformability, several methods were used. Prolonged engagement and reviewing handwritten notes by participants to get feedback from them increased the credibility of the research. Some sections of the transcriptions and extracted codes were sent to external observers to confirm dependability. For facilitating conformability, the research team members discussed the content of the study until consensus was reached.

## Results

Seven experts participated in this study in three separate sessions. Participants had a mean age of 51.7 yr (range 46–63) and mean experience in PHC of 22.3 yr (range 17–34). They were six males and one female; 100% of participants had higher education in medical sciences (pediatrician, psychiatrist, social medicine, pathologist, hospital specialists and Ph.D. in health services management). After codification and analyzing data, eight main categories including quality improvement, management and leadership, community involvement and customer participation, effectiveness of PHC, human resource development, safety, health care evaluation and audit, and health information management were extracted (Table 1). MAXQDA software makes it possible to determine the percentage of each theme by calculating the frequency of codes.

### Quality Improvement

The highest rate (33%) of topics brought out in FGDs were about quality improvement in PHC. They expressed some components for this pillar such as quality management systems, acceptability of service, coordination of care, national regulations for improving service quality, appropriateness of service, existence of reminders in the treatment plans of patients, follow-up in the treatment program of patients, service benchmarking, continuity of care, accessibility and timeliness of service.

Health service should be acceptable, appropriate, continuous and timely. “If a service is not acceptable to the local community, people say no service is provided. Before service delivery, a cultural background must be created among people to accept service” (P2). All participants agreed that access is one of the elements that should be placed in the PHC governance model. “We can see accessibility among PHC principles; we have three types of accessibility: cultural, physical and financial. Cultural accessibility implies that there should be suitable service for all ethnic, religious and social beliefs. Physical means not more than an hour or two hours hike needed to get the service. Financial accessibility means that service should be affordable and people must be able to pay service fee using insurance or anything else” (P1). One of the participants pointed to the role of benchmarking in improving the quality of services in PHC is: “Sometimes it may be necessary to take a look around to find the best actions that would improve the performance of our organization and understand different experiences of countries about the secrets of their achievements” (P5).

### Management and Leadership

The management and leadership category and its subsets comprised 27.7% of the suggested topics in the FGDs. Its subsets are accountability, organizing the PHC system, planning based on local and national priorities, teamwork and communications, development of organizational culture, resource management, strategic planning, and operational planning. Most participants agreed that to improve accountability, the priorities and needs of the local community must be determined and then the individual needs assessment programs should be adjusted. “Accountability of health services organizations is defined as taking into account community and customer interests and expectations. Customers of PHC systems are different from other levels in the health care system. If we want to do any action in the system, customers and other stake-holders must be identified accurately” (P4). Some of the participants considered organizational culture as a critical component of governance model. “Development of organizational culture is very important; it will be a hidden curriculum, organizational behavior that basically has not been written in the job description” (P1). Some participants also stressed the importance of strategic management in every quality program. “Health system administrators have neglected quality at this level because they do not pay attention to quality issues in targeting and strategic planning” (P2). “Sometimes organizations are forced to write the strategic plan, but without operating it, the organization will not take any

### Community Involvement and Customer participation

This made up 12.3% of the FGDs’ content. This pillar’s components are defining stakeholders, service receivers and their participation, privacy and confidentiality of customer information, development of self-care in the community, customer and community empowerment, complaints systems and customer participation in the planning, receiving and evaluation of services.

All participants stressed the need to provide adequate training and empowering people to develop their self-care. “When we talk about community involvement, we are working with words such as education and empowerment of people. It means enabling individuals to protect themselves and their families” (P2). “According to experience of my work in PHC, social participation in health activities needs sensitivity to these issues and it is generated only by providing appropriate training and awareness” (P7). Another participant said empowerment of customers should be reflected at all stages of planning and evaluation: “Our customers should be so empowered that they can participate in all phases of the planning, receiving and evaluation of the received services” (P6).

### Effectiveness of PHC

Seven percent of themes expressed in the FGDs were about effectiveness. Its subsets are access to up-to-date evidence, developing guidelines and protocols, timely check of patients’ care plans and standard treatment plan for patients.

Some participants pointed out the importance of access to evidence and the skill of using evidence to improve quality of service. “For more effective working of health staff, access to the evidence and the ability to using evidence are really important. However, human resources are not educated and skilled for such areas” (P2).

### Human Resource Development

This category and related subsets comprised 7% of the suggested topics in FGDs. The subsets are encouragement and motivation of employees, monitoring and evaluation of staff performance, personal development of employees, continuing education of employees, self-promotion, and self-assessment. Most of the participants believed that a personal development plan is a necessary element of the PHC governance model. “Personal development plans should be placed at the head of any quality improvement programs. This program has led to identifying of the strengths and weaknesses of individuals and planning to resolve these weaknesses, and ultimately growth and excellence in all aspects of the organization” (P4).

One of the participants also believed that the continuing education of health care staff could be effective in improving quality of service: “Health professionals by using programs such as continuing education can gain sufficient knowledge and experience while increasing the efficiency of their job. These programs can guarantee the success of any quality improvement program” (P6).

### Safety

This category and related subsets comprised 5% of the suggested topics in FGDs. its components are safe living environment for residents, employee safety, patient safety, side effects of interventions, safety in service delivery and risk management.

Patients’ and staff’s safety must be considered important components of safety. “In health care, the quality of delivered services and patient safety are closely related. Safety of care is important because a significant percentage of patients get hurt when they receive health care services. Physical and financial costs of these damages are so amazing, but with a little attention can be managed and prevented. Potentially unsafe activities are threatening health care workers, too. I think we can generally put the safety of employees in the subset of safety” (P2). All of the participants agreed with providing a risk management system in PHC. “Similar to hospitals context, establishment of risk management practices in Iran’s PHC system is crucial, including identifying and measuring risks, prevention of them and learning from the experiences of occurred incidents”. (P7)

### Health care evaluation and audit

Five percent of subjects discussed in FGDs were about evaluation and health care audit. This pillar’s components consist of PHC accreditation, performance monitoring, auditing healthcare services and research and development in the health care system. All participants agreed that a qualified system requires activities such as monitoring, audit, and accreditation to confirm movement towards quality. “The PHC system should be continuously monitored to ensure proper performance. Periodic monitoring services at regular intervals will cause ensuring the integrity of the program and achieving the organization’s goals” (P3).

Some participants pointed out that one of the essential elements in the establishment of PHC governance is research and development (R&D). “One of the features of a qualified system or qualified organization is R&D, and its place in our PHC system is empty. R&D is a key element in each health organization. Research involves investigating all the latest technologies that are relevant to the mission of organization. Development is the process of applying appropriate and relevant technologies (P4).

### Health Information Management

Health information management and its subsets comprised 3% of the suggested topics in FGDs including determining performance indicators for various levels of health service, providing evidence needed for managerial decisions, appropriate collection and dissemination of health information and reporting systems.

Some of the participants emphasized the importance of identifying appropriate performance indicators on one hand, and accurate gathering and disseminating of health information on the other hand. “In the absence of performance indicators for each level of service, an information system cannot be able to improve the quality of care” (P2). “The problem seen at all levels of services is the lack of adequate skills at collection, analysis and dissemination of information. Staff training should consider these skills and it will be also in the ability of any information system” (P6).

## Discussion

Overall, in this study, eight main themes and fifty- one sub-themes were identified. Extracted themes were quality improvement, management and leadership, community involvement and customer participation, effectiveness of PHC, human resource development, safety, health care evaluation and audit and health information management.

Some pillars of Australia’s PHC governance model such as education, training and continuing professional development, use of information, clinical audit, clinical effectiveness, and research and patient involvement were similar to pillars proposed for Iran’s PHC governance model. Other pillars of Australia’s PHC governance model such as risk management and staff management exist as subsets of the safety, management and leadership pillars of Iran’s proposed PHC governance model (14). Moreover, in the United Kingdom’s PHC governance model, pillars like clinical effectiveness, provider education and development and clinical audit were similar to pillars proposed for Iran’s PHC governance model. Other pillars of the United Kingdom’s model such as risk management, research and development exist as subsets of the safety, evaluation and health care audit pillars of the Iranian model. The quality improvement pillar in our model is similar to the element of quality assurance in the PHC governance model of UK (9). New Zealand’s PHC governance model is also very similar to Iranian model. Pillars like quality improvement, community involvement and customer participation, effectiveness of PHC, human resource development and safety in our model can be seen as different titles in New Zealand model (6). In the proposed PHC governance model of Iran, as in the model of developed countries, most elements of the WHO model are repeated. Developed countries focused on specific pillars of WHO’s model based on their country’s specific challenges and weaknesses.

In this study quality improvement, management and leadership and community involvement, and customer participation were most repeatable themes in FGD sessions.

Quality improvement is the basic principle of primary health care. This is the everyday task of health care practitioners and the legal obligation of each government. There are many reasons for quality improvement in primary health care, such as increasing accountability of officials, improving efficiency, detecting and reducing adverse events while improving health outcomes and creating correspondence between wants and needs of the customers/patients in health care (15). In Iran, official documents such as “Iranian 20-year vision plan, fourth and fifth economic, social and cultural development programs, and health general policies have emphasized the importance of protecting and promoting public health, including the strengthening and improvement of quality in primary health care (16).

The Iranian primary health care system has some problems in quality improvement such as absence of supportive mechanisms to improve service quality (12), severe problems in terms of continuity and comprehensiveness of services (17) and lack of coordination in various parts of the PHC system (18, 19). These weaknesses confirm experts’ comments about the necessity of pillars related to quality in the governance of Iran’s primary health care system.

Leadership and management are vital elements to control each quality improvement program, such as PHC governance. In fact, the importance of leadership is related to showing the direction of the organization and the roles and responsibilities of individuals and stakeholders inside and outside the organization (20). One of the requirements for the implementation of clinical governance is excellent leadership and management, highly valued staff and active partnership between staff and patients (21).

Given the importance of leadership and management in primary health care governance, Iran’s PHC system faces some weaknesses in this pillar such as lack of accountability (11)(6, 7), weaknesses in resource management (financial, human resource, equipment and physical space), unstable life of management, failure to select managers based on merit, and lack of strategic management within organizations (11, 22).

The third important pillar from the perspective of the participants in this study is “community involvement and customer participation.” Involving community in health system leads to restoration of public trust, improved health outcomes, appropriate use of health services and higher cost-effectiveness (23). Community and customer participation in primary health care governance in most countries is underdeveloped and public involvement has a low priority in health systems due to limited used resources and knowledge (24).

Although one of the principles of primary health care is community involvement, this important matter has not been addressed in the primary health care system of Iran (19). Some problems of involving community are lack of ownership sense in society toward the health system and communities’ low confidence about services (25). In this study, by doing the measures like defining stakeholders, guaranteeing privacy and confidentiality of customer information, assessing needs of the local community and identifying local health priorities, empowerment of customer and community, development of self-care in the community and establishing complaint management systems, concrete steps to improve community involvement and customer participation can be taken.

The FGDs participants are limited to local experts in Tabriz medical sciences university. Because of distance between Tabriz and other capital cities, particularly Tehran, and also occupancy of experts in this area we had to use the local experts only. Moreover, researchers could not conduct a pilot study to examine the feasibility of pillars and subsets. The suggested concepts would be implemented in one of district health centers during next years.

## Conclusion

Elements that suggested for designing Iran’s PHC governance model are presented according to internal informed expert’s opinions and taking into account Iran’s PHC system real status so they have potential to improve the quality and accountability of the PHC system. Attitudes and behavior of managers and caregivers will be change and they will be ready for any necessary qualitative transformation, by implementing these elements to Iran’s PHC system. Iran’s PHC managers can use the results of this study to establish their evaluation system. Moreover, they can define proper performance indicators for each pillar and elements, so it will be possible assessing the progress of the PHC system towards excellence and it will prevent any mental judgments about system performance.

## Ethical considerations

Ethical issues (Including plagiarism, informed consent, misconduct, data fabrication and/or falsification, double publication and/or submission, redundancy, etc.) have been completely observed by the authors.
